# Supplementary material for: Ecological indicators reveal historical regime shifts in the Black Sea ecosystem
Source: PeerJ. 2023 Jul 11;11:e15649. doi: 10.7717/peerj.15649 (PMC10348305; doi:10.7717/peerj.15649)
Supplement: Supplemental Information 1 [file peerj-11-15649-s001.docx]

| **Functional Groups** | **Original value** | **Calibrated value** | **Sources** |
| --- | --- | --- | --- |
| **Dolphins** | | | |
| Biomass | 0.05 | 0.03 | Moiseev (1969) c.f. Ivanov & Beverton (1985) |
| P/B | 0.35 | 0.35 | Moiseev (1969) c.f. Ivanov & Beverton (1985) |
| Q/B | 17.05 | 17.05 | Empirical equation Innes et al. (1987) using average weights from Ivanov & Beverton (1985) |
| Diet | Santos et al. (2001), Börjesson, Berggren & Ganning (2003), Pierrepont et al. (2005) | | |
| **Atlantic bonito** | | | |
| Biomass | 0.0593 | 0.0593 | Ivanov & Beverton (1985) assuming a median biomass value |
| P/B | 0.825 | 0.825 | Z = F + M; hence, empirical equation for M by Pauly (1980) using length-weight relationship and L_∞_ from Kahraman et al. (2014), F from Daskalov et al. (2020) |
| Q/B | 7.549 | 7.549 | Empirical equation by Palomares & Pauly (1998) using length-weight relationship and L_∞_ from Kahraman et al. (2014) |
| Diet | Genç, Başçınar & Dağtekin (2019) | | |
| **Bluefish** | | | |
| Biomass | 0.0124 | 0.0149 | Ivanov & Beverton (1985) assuming a biomass about quintuple of average catch in the 1960s |
| P/B | 0.960 | 0.960 | Akyol & Ceyhan (2007) |
| Q/B | 5.109 | 5.109 | Empirical equation by Palomares & Pauly (1998) using length-weight relationship from Kalaycı et al. (2007) and L_∞_ from Ceyhan et al. (2007) |
| Diet | Bal, Yanik & Türker (2020) | | |
| **Atlantic mackerel** | | | |
| Biomass | 0.0201 | 0.0201 | Prodanov et al. (1997) |
| P/B | 1.425 | 1.425 | Prodanov et al. (1997) |
| Q/B | 8.665 | 8.665 | Empirical equation by Palomares & Pauly (1998) using length-weight and L_∞_ parameters from Bal & Türker (2019) |
| Diet | Greenstreet (1996) c.f. Froese & Pauly (2023) | | |
| **Whiting** | | | |
| Biomass | 0.223 | 0.223 | Prodanov et al. (1997) |
| P/B | 0.915 | 0.915 | Prodanov et al. (1997) |
| Q/B | 5.072 | 5.072 | Empirical equation by Palomares & Pauly (1998) using W_∞_ from Prodanov (1980) |
| Diet | Mazlum & Bilgin (2014) | | |
| **Turbot** | | | |
| Biomass | 0.0162 | 0.0162 | Prodanov et al. (1997) |
| P/B | 0.551 | 0.551 | Prodanov et al. (1997) |
| Q/B | 2.759 | 2.759 | Empirical equation by Palomares & Pauly (1998) using length-weight relationship from Samsun, Kalaycı & Samsun (2007) and L_∞_ from Özdemir, Erdem & Sümer (2006) |
| Diet | Zengin (2000) | | |
| **Red mullet** | | | |
| Biomass | 0.0034 | 0.013 | Calculated using median F values from Kasapoğlu (2018), Aydın & Karadurmuş (2013) and catch value from Ivanov & Beverton (1985) |
| P/B | 1.66 | 1.66 | Kasapoğlu (2018) |
| Q/B | 7.245 | 7.245 | Empirical equation by Palomares & Pauly (1998) using length-weight and L_∞_ from Kasapoğlu (2018) |
| Diet | Onay & Dalgic (2019) | | |
| **Spiny dogfish** | | | |
| Biomass | 0.0587 | 0.0587 | Prodanov et al. (1997) |
| P/B | 0.164 | 0.164 | Prodanov et al. (1997) |
| Q/B | 2.872 | 2.872 | Empirical equation by Palomares & Pauly (1998) using W_∞_ from Avsar (2001) |
| Diet | Demirhan, Seyhan & Basusta (2007) | | |
| **Mediterranean horse mackerel** | | | |
| Biomass | 0.191 | 0.191 | Prodanov et al. (1997) |
| P/B | 1.712 | 1.712 | Samsun, Akyol & Ceyhan (2018) |
| Q/B | 7.375 | 7.375 | Empirical equation Palomares & Pauly (1998) using length-weight relationship and L_∞_ from Kasapoğlu (2018) |
| Diet | Georgieva et al. (2019) | | |
| **Pontic shad** | | | |
| Biomass | 0.00815 | 0.00815 | Prodanov et al. (1997) |
| P/B | 1.28 | 1.28 | Özdemir et al. (2018) |
| Q/B | 6.4 | 6.4 | Empirical equation by Palomares & Pauly (1998) using length-weight relationship and L_∞_ from Özdemir et al. (2018) |
| Diet | Mazlum & Akgumus (2019) | | |
| **Sprat** | | | |
| Biomass | 0.296 | 0.412 | Prodanov et al. (1997) |
| P/B | 1.7 | 1.7 | Prodanov et al. (1997) |
| Q/B | 10.58 | 10.58 | Empirical equation by Palomares & Pauly (1998) using length-weight relationship from Satilmis et al. (2014) and L_∞_ from Stoyanov (1965) |
| Diet | Bayhan & Sever (2015) | | |
| **Anchovy 1,1+** | | | |
| Biomass | 0.597 | 0.597 | Prodanov et al. (1997) |
| P/B | 1.807 | 1.807 | Z = F + M; hence, F from Prodanov et al. (1997) and M from Ivanov & Beverton (1985) |
| Q/B | 9.249 | 9.249 | Empirical equation by Palomares & Pauly (1998) using length-weight relationship from Satilmis et al. (2014) and L_∞_ from Karacam & Düzgünes (1990) |
| Diet | Bulgakova (1996) | | |
| **Anchovy 0,0+** | | | |
| Biomass | - | - | Estimated by Ecopath |
| P/B | 6.44 | 6.44 | Oguz, Salihoglu & Fach (2008) |
| Q/B | - | - | Estimated by Ecopath |
| Diet |  |  | Bulgakova (1996) |
| K | 0.324 | 0.324 | Karacam & Düzgünes (1990) |
| W_maturity_/W_∞_ | 0.135 | 0.135 | Calculated based on W∞ from Karacam & Düzgünes (1990)and L_50_ from Samsun, Samsun & Karamollaoğlu (2004) |
| **Benthic invertebrates** | | | |
| Biomass | 0.75 | 0.75 | Ivanov & Beverton (1985) |
| P/B | 2.5 | 2.5 | Moiseev (1969) c.f. Ivanov & Beverton (1985) |
| Q/B | 22.99 | 22.99 | Calculated using temperature correction factor as per Opitz (1996) using the deep-basin averaged sea surface temperature of 15°C between 1960-1999 that was adjusted considering model’s overestimation with comparison to Pathfinder data (Miladinova et al., 2017) |
| Diet | Tsagarakis et al. (2010) | | |
| **Aurelia** | | | |
| Biomass | 0.0684 | 0.0484 | Shiganova et al. (2008) |
| P/B | 10.95 | 10.95 | Daskalov (2002) |
| Q/B | 34.76 | 34.76 | based on P/Q (0.315) by Olesen, Frandsen & Riisgård (1994) |
| Diet | Anninsky et al. (2020) | | |
| ***Beroe ovata*** | | | |
| Biomass | - | - | None in 1960 |
| P/B | 11.72 | 11.72 | Based on P/Q = 0.3 by Finenko et al. (2003) |
| Q/B | 39.05 | 39.05 | Kideys et al. (2004) |
| Diet | Kamburska (2004), Berdnikov et al. (1999) | | |
| ***Mnemiopsis leidyi*** | | | |
| Biomass | - | - | None in 1960 |
| P/B | 20.1 | 20.1 | Shiganova et al. (2018) |
| Q/B | 55.68 | 55.68 | Based on P/Q = 0.361 by Reeve, Syms & Kremer (1989) |
| Diet | Mutlu (1999) | | |
| ***Pleurobrachia pileus*** | | | |
| Biomass | 0.01 | 0.01 | Shiganova et al. (2008) |
| P/B | 10.95 | 10.95 | Daskalov (2002) |
| Q/B | 29.2 | 29.2 | Daskalov (2002) |
| Diet | Mazlum et al. (2018) | | |
| ***Noctiluca scintillans*** | | | |
| Biomass | 0.09 | 0.11 | Daskalov (2002) |
| P/B | 7.3 | 7.3 | Greze (1979) |
| Q/B | 36.2 | 36.2 | Daskalov (2002) |
| Diet | Berdnikov (1999) | | |
| **Zooplankton** | | | |
| Biomass | 0.4 | 0.4 | Shiganova et al. (2008) |
| P/B | 30 | 38 | Datzko (1954) and Vodyanitzki (1956) c.f. Ivanov & Beverton (1985) |
| Q/B | 152 | 152 | Based on P/Q = 0.25 from Straile (1997) |
| Diet | Daskalov (2002) | | |
| **Phytoplankton** | | | |
| Biomass | 0.25 | 0.6 | Yunev (2011) |
| P/B | 252 | 105 | Calculated to match 63+-18 gC/m2/y annual primary production as per (Yunev, 2011) |
| **Detritus** | | | |
| Biomass | 5.926 | 5.926 | Empirical equation by Pauly et al. (1993) using 63 gC/m2/y primary production from Yunev (2011) and euphotic zone depth of 50 m (Zenkevitch, 1963) |

References

Akyol O, Ceyhan T. 2007. Exploitation and mortalities of bluefish (Pomatomus saltatrix L.) in the Sea of Marmara, Turkey. *Journal of Applied Biological Sciences* 1:25–27.

Anninsky B, Finenko G, Datsyk N, Kıdeyş A. 2020. Trophic ecology and assessment of the predatory impact of the Moon jellyfish Aurelia aurita (Linnaeus, 1758) on zooplankton in the Black Sea. *Cahiers de Biologie Marine* 61.

Avsar D. 2001. Age, growth, reproduction and feeding of the spurdog (Squalus acanthias Linnaeus, 1758) in the South-eastern Black Sea. *Estuarine, Coastal and Shelf Science* 52:269–278.

Aydın M, Karadurmuş U. 2013. An investigation on age, growth and biological characteristics of red mullet (Mullus barbatus ponticus, Essipov, 1927) in the Eastern Black Sea.

Bal H, Türker D. 2019. Investigation some biological properties of Atlantic mackerel Scomber scombrus Linnaeus, 1758 in the Sea of Marmara. *Natural and Engineering Sciences* 4:133–140.

Bal H, Yanik T, Türker D. 2020. Diet composition of bluefish Pomatomus saltatrix (Linnaeus, 1766) in the Sea of Marmara. *Marine Science and Technology Bulletin* 9:46–50.

Bayhan B, Sever TM. 2015. Spring diet and feeding strategy of the European sprat Sprattus sprattus (L., 1758) from the Black Sea coast of Turkey. *Turkish Journal of Agriculture-Food Science and Technology* 3:697–700.

Berdnikov SV, Selyutin VV, Vasilchenko VV, Caddy JF. 1999. Trophodynamic model of the Black and Azov Sea pelagic ecosystem: consequences of the comb jelly, Mnemiopsis leydei, invasion. *Fisheries Research* 42:261–289.

Börjesson P, Berggren P, Ganning B. 2003. Diet of harbor porpoises in the Kattegat and Skagerrak seas: accounting for individual variation and sample size. *Marine Mammal Science* 19:38–058.

Bulgakova YV. 1996. Feeding in the Black Sea anchovy: diet composition, feeding behaviour, feeding periodicity and daily rations. *Scientia Marina* 60:283–284.

Ceyhan T, Akyol O, Ayaz A, Juanes F. 2007. Age, growth, and reproductive season of bluefish (Pomatomus saltatrix) in the Marmara region, Turkey. *ICES Journal of Marine Science* 64:531–536.

Daskalov GM. 2002. Overfishing drives a trophic cascade in the Black Sea. *Marine Ecology Progress Series* 225:53–63.

Daskalov GM, Demirel N, Ulman A, Georgieva Y, Zengin M. 2020. Stock dynamics and predator–prey effects of Atlantic bonito and bluefish as top predators in the Black Sea. *ICES Journal of Marine Science* 77:2995–3005.

Datzko VG. 1954. Some chemical indices of the productivity of the Black Sea. *Tr. Vses. Nauchno-Issled. Inst. Morsk. Rybn. Rhoz. Okeanogr* 28:188–202.

Demirhan SA, Seyhan K, Basusta N. 2007. Dietary overlap in spiny dogfish (Squalus acanthias) and thornback ray (Raja clavata) in the southeastern black sea. *Ekoloji* 16:1–8.

Finenko GA, Romanova ZA, Abolmasova GI, Anninsky BE, Svetlichny LS, Hubareva ES, Bat L, Kideys AE. 2003. Population dynamics, ingestion, growth and reproduction rates of the invader Beroe ovata and its impact on plankton community in Sevastopol Bay, the Black Sea. *Journal of Plankton research* 25:539–549.

Froese R, Pauly D (eds.). 2023. FishBase. World Wide Web electronic publication.

Genç Y, Başçınar NS, Dağtekin M. 2019. Feeding habits during migration of the Atlantic bonito Sarda sarda (Bloch, 1793) to the Black Sea. *Marine Biology Research* 15:125–136.

Georgieva YG, Daskalov GM, Klayn SL, Stefanova KB, Stefanova ES. 2019. Seasonal diet and feeding strategy of horse mackerel Trachurus mediterraneus (Steindachner, 1868)(Perciformes: Carangidae) in the south-western Black Sea. *Acta Zool. Bulg* 71:201–210.

Greenstreet SPR. 1996. Estimation of the daily consumption of food by fish in the North Sea in each quarter of the year. *Scottish Fish. Res. Rep*.

Innes S, Lavigne DM, Earle WM, Kovacs KM. 1987. Feeding rates of seals and whales. *The Journal of Animal Ecology*:115–130.

Ivanov L, Beverton RH. 1985. The fisheries resources of the Mediterranean, part two: Black Sea. *Etudes et Revues du Conseil général des Pêches pour la Méditerranée* 60.

Kahraman AE, Göktürk D, Yildiz T, Uzer U. 2014. Age, growth, and reproductive biology of Atlantic bonito (Sarda sarda Bloch, 1793] from the Turkish coasts of the Black Sea and the Sea of Marmara. *Turkish Journal of Zoology* 38:614–621.

Kalaycı F, Samsun N, Bilgin S, Samsun O. 2007. Length-weight relationship of 10 fish species caught by bottom trawl and midwater trawl from the Middle Black Sea, Turkey. *Turkish Journal of Fisheries and Aquatic Sciences* 7.

Kamburska L. 2004. Effects of Beroe cf ovata on gelatinous and other zooplankton along the Bulgarian Black Sea Coast. In: *Aquatic Invasions in the Black, Caspian, and Mediterranean Seas: The Ctenophores Mnemiopsis leidyi and Beroe in the Ponto-Caspian and other Aquatic Invasions*. Springer Netherlands, 137–154.

Karacam H, Düzgünes E. 1990. Age, growth and meat yield of the European anchovy (Engraulis encrasicolus, L. 1758) in the Black Sea. *Fisheries Research* 9:181–186.

Kasapoğlu N. 2018. Age, growth, and mortality of exploited stocks: anchovy, sprat, Mediterranean horse mackerel, whiting, and red mullet in the southeastern Black Sea. *Aquatic Sciences and Engineering* 33:39–49.

Kideys AE, Finenko GA, Anninsky BE, Shiganova TA, Roohi A, Tabari MR, Youseffyan M, Rostamian MT, Rostami H, Negarestan H. 2004. Physiological characteristics of the ctenophore Beroe ovata in Caspian Sea water. *Marine Ecology Progress Series* 266:111–121.

Mazlum R, Akgumus S. 2019. Stomach content analysis and length-weight relationship of the Pontic shad Alosa immaculata Bennett, 1835 (Pisces: Clupeidae), from the eastern Black Sea coast of Turkey. *Indian Journal of Fisheries* 66.

Mazlum RE, Bilgin S. 2014. Age, growth, reproduction and diet of the whiting, Merlangius merlangus euxinus (Nordmann, 1840. In: *in the southeastern Black Sea*.

Miladinova S, Stips A, Garcia-Gorriz E, Macias Moy D. 2017. Black Sea thermohaline properties: Long-term trends and variations. *Journal of Geophysical Research: Oceans* 122:5624–5644. DOI: 10.1002/2016JC012644.

Moiseev PA. 1969. The living resources of the world oceans. :338.

Mutlu E. 1999. Distribution and abundance of ctenophores and their zooplankton food in the Black Sea. II. *Mnemiopsis leidyi. Marine Biology* 135:603–613.

Oguz T, Salihoglu B, Fach B. 2008. A coupled plankton–anchovy population dynamics model assessing nonlinear controls of anchovy and gelatinous biomass in the Black Sea. *Marine Ecology Progress Series* 369:229–256.

Olesen NJ, Frandsen K, Riisgård HU. 1994. Population dynamics, growth and energetics of jellyfish Aurelia aurita in a shallow fjord. *Marine Ecology Progress Series*:9–18.

Onay H, Dalgic G. 2019. Seasonal changes in the food spectrum and day-time rhythm of feeding in red mullet Mullus barbatus (Linnaeus, 1758) in the southeast Black Sea. *Fresius Environmental Bulletin* 28:2671–2678.

Opitz S. 1996. Trophic interactions in Caribbean coral reefs. 1085.

Özdemir S, Erdem Y, Sümer Ç. 2006. The comparison of population parameters of turbot (Psetta maxima, Linneaus, 1758) and whiting (Merlangius merlangus euxinus, Nordman 1840) which are estimated by using age and length data. *Ondokuz Mayıs University Journal of Faculty of Agriculture* 21:71–75.

Özdemir S, Söyleyici H, Birinci Özdemir Z, Erdem E. 2018. Estimation of Growth and Population Parameters using Age and Length Composition of Allis shad (Alosa immaculata Bennett, 1835) captured in the Black Sea. *Süleyman Demirel Üniversitesi Eğirdir Su Ürünleri Fakültesi Dergisi* 14:102–112.

Palomares MLD, Pauly D. 1998. Predicting food consumption of fish populations as functions of mortality, food type, morphometrics, temperature and salinity. *Marine and freshwater research* 49:447–453.

Pauly D. 1980. On the interrelationships between natural mortality, growth parameters, and mean environmental temperature in 175 fish stocks. *ICES journal of Marine Science* 39:175–192.

Pierrepont JF, Dubois B, Desormonts S, Santos MB, Robin JP. 2005. Stomach contents of English Channel cetaceans stranded on the coast of Normandy. *Journal of the Marine Biological Association of the United Kingdom* 85:1539–1546.

Prodanov K. 1980. Preliminary age-and growth data on Black Sea whiting, Odontogadus merlangus euxinus Nordmann, off Bulgarian Black Sea coast. In: *Proceedings of the Institute of Fisheries-Varna*. 121–134.

Prodanov K, Mikhaylov K, Daskalov G, Maxim K. 1997. Environmental management of fish resources in the Black Sea and their rational exploitation. *Gen. Fish. Counc. Mediterr. Stud. Rev* 68:178.

Reeve MR, Syms MA, Kremer P. 1989. Growth dynamics of a ctenophore (Mnemiopsis) in relation to variable food supply. I. Carbon biomass, feeding, egg production, growth and assimilation efficiency. *Journal of Plankton Research* 11:535–552.

Samsun O, Akyol O, Ceyhan T. 2018. Mortalities and exploitation rate of mediterranean horse mackerel, Trachurus mediterraneus (Steindachner, 1868) in the Central Black Sea. *Turkish Journal of Maritime and Marine Sciences* 4:139–145.

Samsun N, Kalaycı F, Samsun O. 2007. Seasonal variation in length, weight, and sex distribution of turbot (Scophthalmus maeoticus Pallas, 1811) in the Sinop region (Black Sea) of Turkey. *Turkish Journal of Zoology* 31:371–378.

Samsun O, Samsun N, Karamollaoğlu AC. 2004. Age, growth, and mortality rates of the European anchovy (Engraulis encrasicolus L. 1758) off the Turkish Black Sea coast. *Turkish Journal of Veterinary & Animal Sciences* 28:901–910.

Santos MB, Pierce GJ, Reid RJ, Patterson IAP, Ross HM, Mente E. 2001. Stomach contents of bottlenose dolphins (Tursiops truncatus) in Scottish waters. *Journal of the Marine Biological Association of the United Kingdom* 81:873–878.

Satilmis HH, Sumer C, Ozdemir S, Bayrakli B. 2014. Length-weight relationships of the three most abundant pelagic fish species caught by mid-water trawls and purse seine in the Black Sea. *Cahiers de biologie marine* 55:259–265.

Shiganova TA, Alekseenko E, Lidia M, Paul N. 2018. Modelling assessment of interactions in the Black Sea of the invasive ctenophores Mnemiopsis leidyi and Beroe ovata. *Ecological Modelling* 376:1–14.

Shiganova TA, Musaeva E, Arashkevich E, Kamburska L, Stefanova K, Mihneva M, Polishchuk L, Timofte F, Ustun F, Oguz T. 2008. The state of zooplankton. In: Oguz T ed. *State of the Environment of the Black Sea (2001–2006/7). Publications of the Commission on the Protection of the Black Sea Against Pollution (BSC) 2008-3*. Istanbul, Turkey, 201–246.

Stoyanov S. 1965. Dynamics of the Black Sea sprat stock Sprattus sprattus sulinus (Antipa. In: *Proc. of the Research Institute of Fisheries and Oceanography–Varna*. 21–47.

Straile D. 1997. Gross growth efficiencies of protozoan and metazoan zooplankton and their dependence on food concentration, predator‐prey weight ratio, and taxonomic group. *Limnology and Oceanography* 42:1375–1385.

Tsagarakis K, Coll M, Giannoulaki M, Somarakis S, Papaconstantinou C, Machias A. 2010. Food-web traits of the North Aegean Sea ecosystem (Eastern Mediterranean) and comparison with other Mediterranean ecosystems. *Estuarine, Coastal and Shelf Science* 88:233–248.

Vodyanitzki VA. 1956. On the problem of biological productivity of the Black Sea. *Tr. Sevastop. Biol. Sın., Akad. Nauk Ukr. S. SR* 8:347–438.

Yunev OA. 2011. Eutrophication and annual primary production of phytoplankton in the deep-water part of the Black Sea. *Oceanology* 51:616–625.

Zengin M. 2000. The Bio˗ ecology, Population Parameters and Stocks Assessment of the Turbot (Scopthalmus maeoticus Pallas, 1811) in the Turkish coast of the Eastern Black Sea. (Doctoral dissertation, Ph. D. Thesis. Thesis. Trabzon: Fen Bilimleri Ensitütüsü, Karadeniz Teknik Üniversitesi.

Zenkevitch LA. 1963. *Biology of the seas of the USSR*. London: Allen &Unwin.
