# Supplementary material for: Ecological indicators reveal historical regime shifts in the Black Sea ecosystem
Source: PeerJ. 2023 Jul 11;11:e15649. doi: 10.7717/peerj.15649 (PMC10348305; doi:10.7717/peerj.15649)
Supplement: Supplemental Information 2 [file peerj-11-15649-s002.docx]

| # | **Prey \ predator** | **1** | **2** | **3** | **4** | **5** | **6** | **7** | **8** | **9** | **10** | **11** | **12** | **13** | **14** | **15** | **16** | **17** | **18** | **19** | **20** |
| --- | --- | --- | --- | --- | --- | --- | --- | --- | --- | --- | --- | --- | --- | --- | --- | --- | --- | --- | --- | --- | --- |
| 1 | Dolphins |  |  |  |  |  |  |  | 0.017 |  |  |  |  |  |  |  |  |  |  |  |  |
| 2 | A. bonito | 0.070 |  |  |  |  |  |  |  |  |  |  |  |  |  |  |  |  |  |  |  |
| 3 | Bluefish | 0.006 | 0.002 | 0.022 |  |  |  |  |  |  |  |  |  |  |  |  |  |  |  |  |  |
| 4 | A. mackerel | 0.040 |  |  |  |  |  |  |  |  |  |  |  |  |  |  |  |  |  |  |  |
| 5 | Whiting | 0.070 | 0.023 | 0.053 |  | 0.100 | 0.619 |  | 0.050 |  |  |  |  |  |  |  |  |  |  |  |  |
| 6 | Turbot |  |  |  |  |  |  |  |  |  |  |  |  |  |  |  |  |  |  |  |  |
| 7 | Red mullet | 0.003 | 0.006 | 0.029 |  | 0.007 | 0.037 | 0.040 | 0.003 |  |  |  |  |  |  |  |  |  |  |  |  |
| 8 | Spiny dogfish |  |  |  |  |  |  |  | 0.000 |  |  |  |  |  |  |  |  |  |  |  |  |
| 9 | Med. horse mackerel | 0.100 | 0.177 | 0.087 |  | 0.136 | 0.081 |  | 0.001 |  | 0.050 |  |  |  |  |  |  |  |  |  |  |
| 10 | Pontic shad | 0.010 | 0.003 |  |  |  | 0.055 |  |  |  |  |  |  |  |  |  |  |  |  |  |  |
| 11 | Sprat | 0.383 | 0.016 | 0.026 |  | 0.210 | 0.189 |  | 0.010 | 0.160 | 0.030 |  |  |  |  |  |  |  |  |  |  |
| 12 | Anchovy 1,1+ | 0.318 | 0.373 | 0.383 |  | 0.346 | 0.003 |  | 0.855 | 0.100 | 0.200 |  |  |  |  |  |  |  |  |  |  |
| 13 | Anchovy 0,0+ |  |  |  |  |  |  |  |  |  |  |  |  |  |  |  |  | 0.001 |  |  |  |
| 14 | Benthic invert. |  |  |  | 0.060 | 0.201 | 0.016 | 0.960 | 0.063 |  |  |  |  |  | 0.050 |  |  |  |  |  |  |
| 15 | A. aurita |  |  |  |  |  |  |  |  |  |  |  |  |  |  |  |  |  |  |  |  |
| 16 | B. ovata |  |  |  |  |  |  |  |  |  |  |  |  |  |  |  |  |  |  |  |  |
| 17 | M. leidyi |  |  |  |  |  |  |  |  |  |  |  |  |  |  |  | 0.500 |  |  |  |  |
| 18 | P. pileus |  |  |  |  |  |  |  |  |  |  |  |  |  |  |  | 0.028 | 0.005 |  |  |  |
| 19 | N. scintillans |  |  |  |  |  |  |  |  |  |  |  |  |  |  | 0.450 | 0.028 |  |  |  |  |
| 20 | Zooplankton |  |  |  | 0.540 |  |  |  |  | 0.740 | 0.720 | 1.000 | 1.000 | 0.800 |  | 0.325 | 0.306 | 0.799 | 0.045 | 0.035 | 0.040 |
| 21 | Phytoplankton |  |  |  |  |  |  |  |  |  |  |  |  | 0.200 |  |  |  |  | 0.746 | 0.345 | 0.725 |
| 22 | Detritus |  |  |  |  |  |  |  |  |  |  |  |  |  | 0.950 | 0.225 | 0.138 | 0.195 | 0.209 | 0.620 | 0.235 |
|  | Import |  | 0.401 | 0.400 | 0.400 |  |  |  |  |  |  |  |  |  |  |  |  |  |  |  |  |
