# Supplementary material for: Ecological indicators reveal historical regime shifts in the Black Sea ecosystem
Source: PeerJ. 2023 Jul 11;11:e15649. doi: 10.7717/peerj.15649 (PMC10348305; doi:10.7717/peerj.15649)
Supplement: Supplemental Information 3 — The best model is the one with the lowest AICc score. [file peerj-11-15649-s003.docx]

| **Calibration Option** | **Minimum SS (**$\boldsymbol{SS=}\sum{\mathbf{ln}\boldsymbol{(}\frac{\boldsymbol{O}_{\boldsymbol{i}}}{\boldsymbol{S}_{\boldsymbol{i}}}\boldsymbol{)}}^{\boldsymbol{2}}$**)** | **AIC_c_** |
| --- | --- | --- |
| Baseline | 1619.3 | 586.2 |
| Baseline and trophic effects (81 *v*) | 1294.2 | 611.1 |
| Baseline and primary production anomaly (5 spline points) | 1563.7 | 571.7 |
| Baseline, trophic effects (81 *v*) and primary production anomaly (5 spline points) | 1230.1 | 588.1 |
| Fishing | 1419.8 | 493.6 |
| Fishing and trophic effects (81 *v*) | 722.3 | 199.9 |
| Fishing and primary production anomaly (5 spline points) | 1325.7 | 455.3 |
| Fishing, trophic effects (81 *v*) and primary production anomaly (5 spline points) | 601.0 | 83.1 |
