# Supplementary figures and images for: Ecological indicators reveal historical regime shifts in the Black Sea ecosystem
Source: PeerJ. 2023 Jul 11;11:e15649. doi: 10.7717/peerj.15649 (PMC10348305; doi:10.7717/peerj.15649)

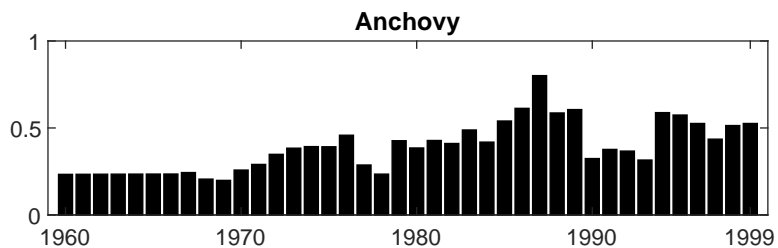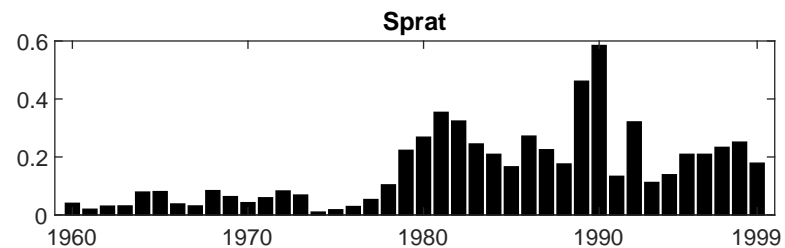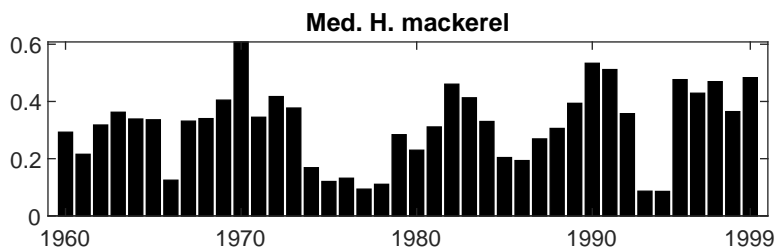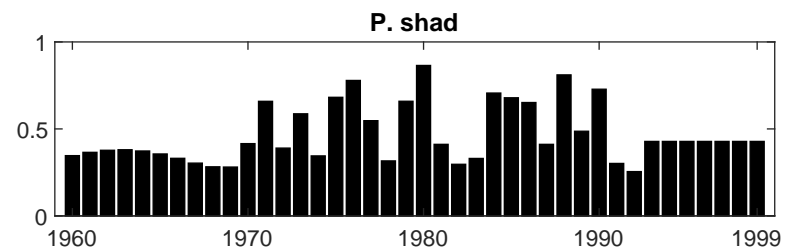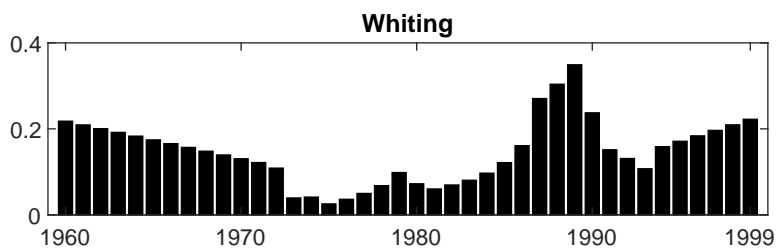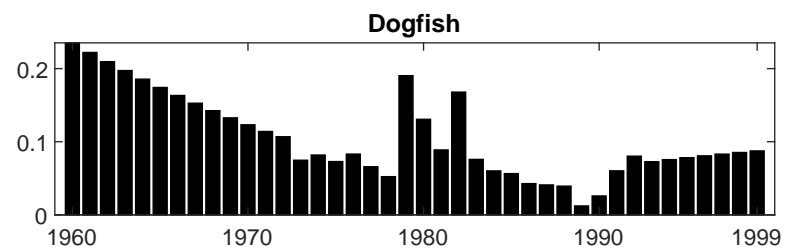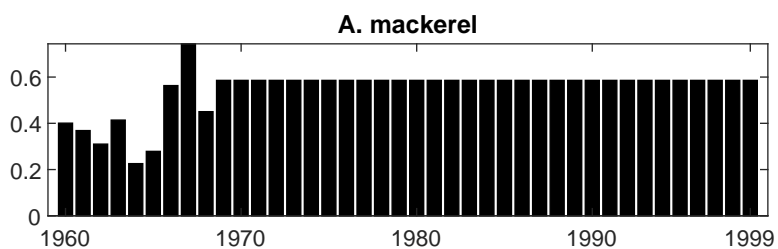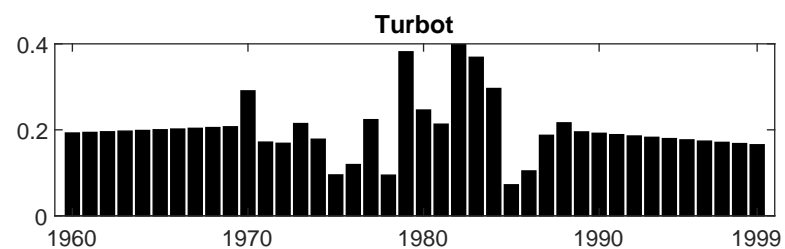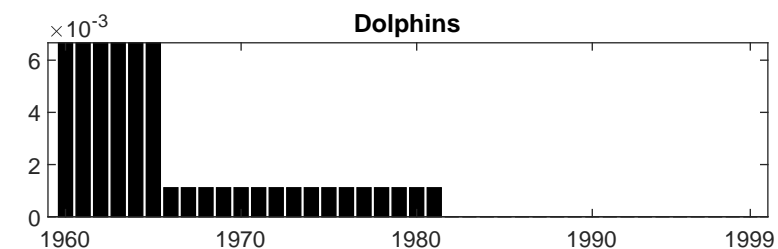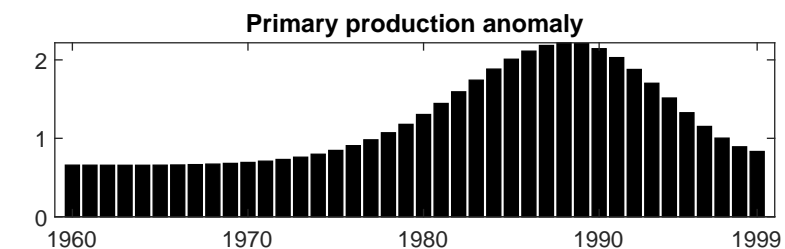

Supplement: Supplemental Information 4 — Note that the dolphins fishery halted due to basin-wide fishing ban in 1982. [file peerj-11-15649-s004.pdf]

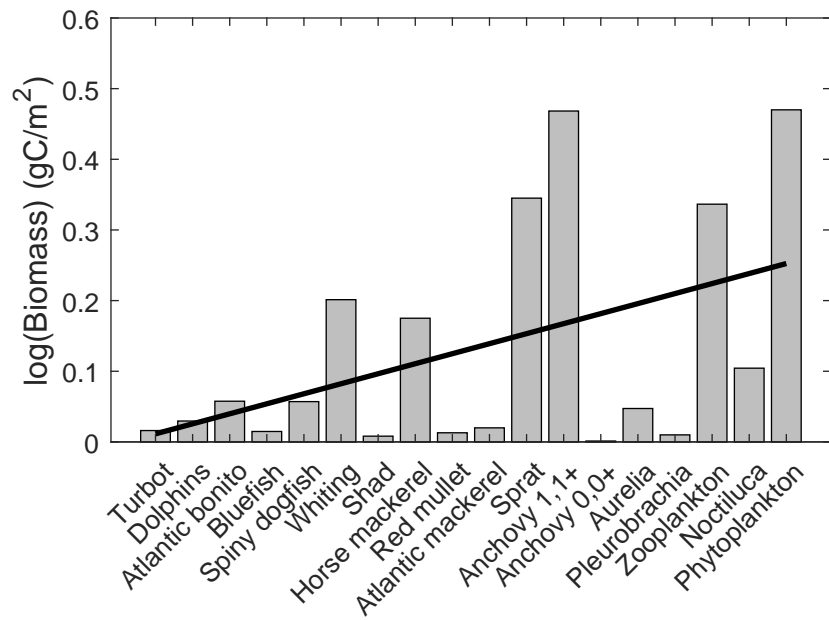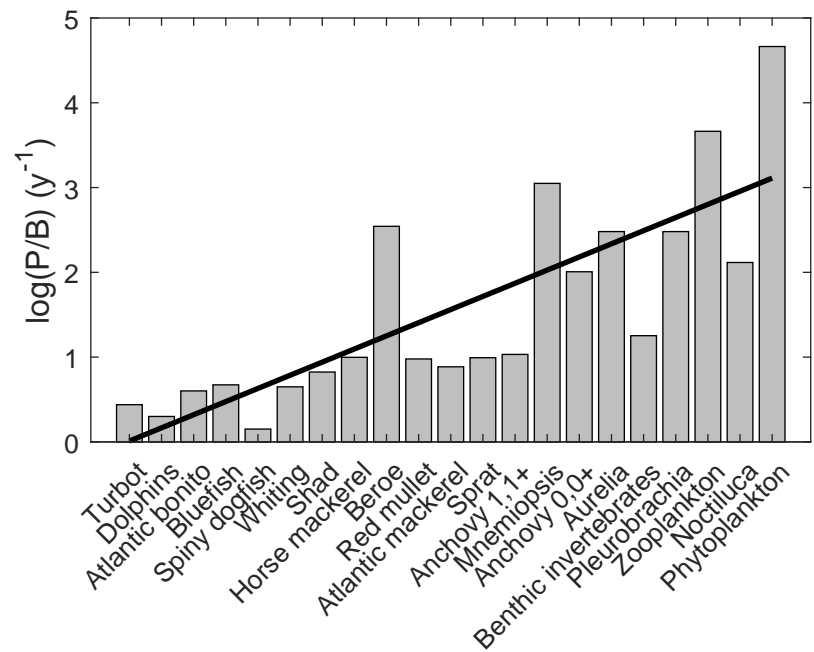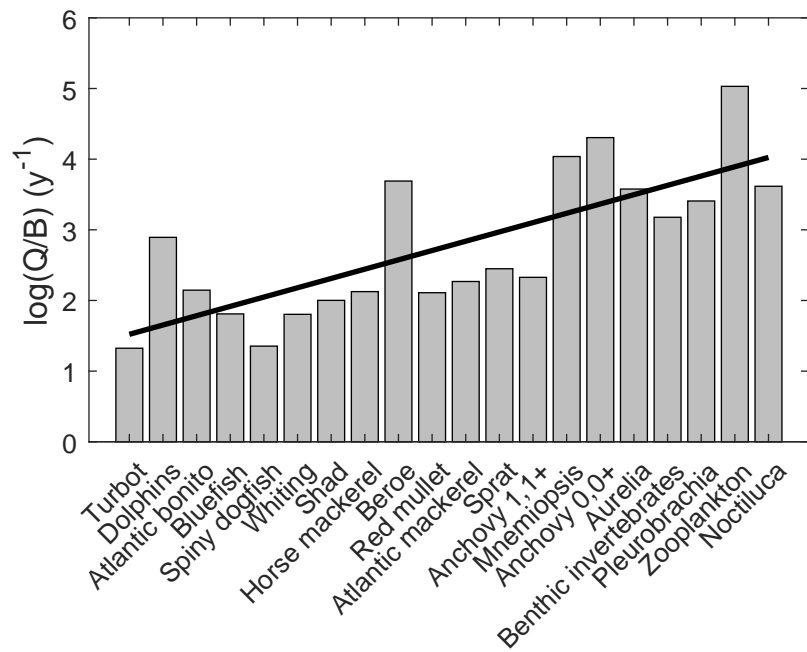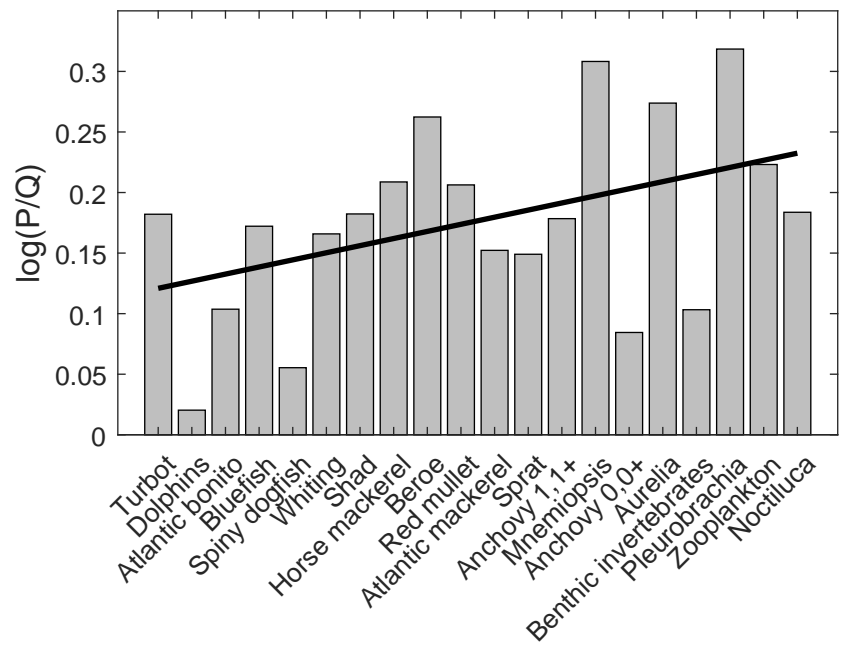

Supplement: Supplemental Information 5 — Bars show the values and black straight lines show the trend of the values. X axes show the functional groups and species sorted from high to low trophic level groups, and species sorted from high to low trophic levels. [file peerj-11-15649-s005.pdf]

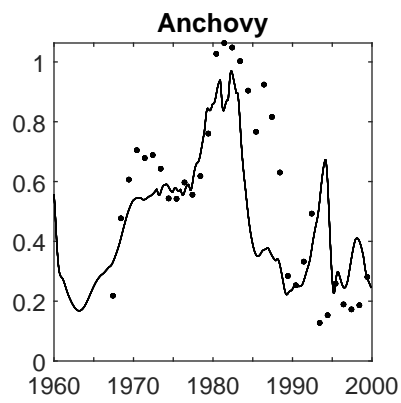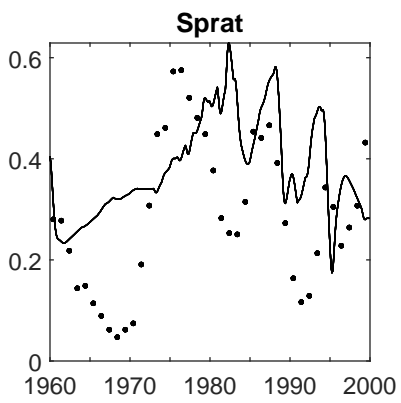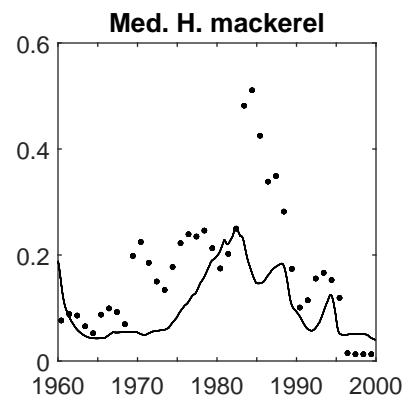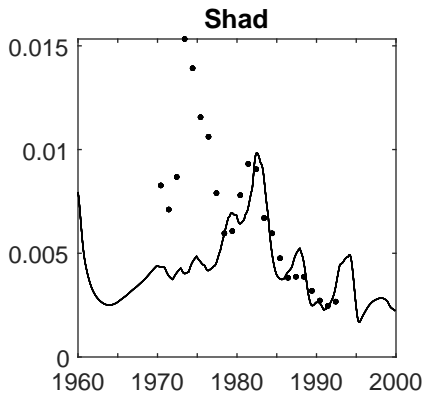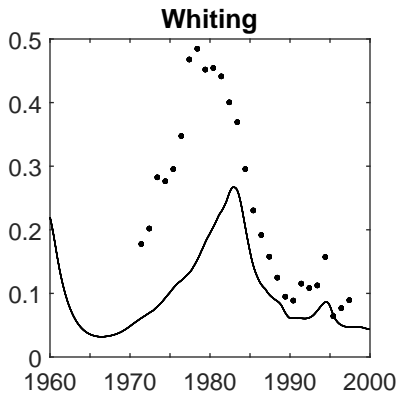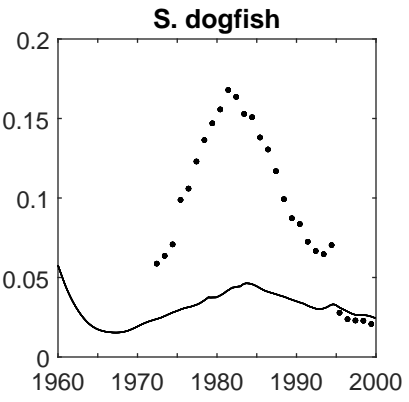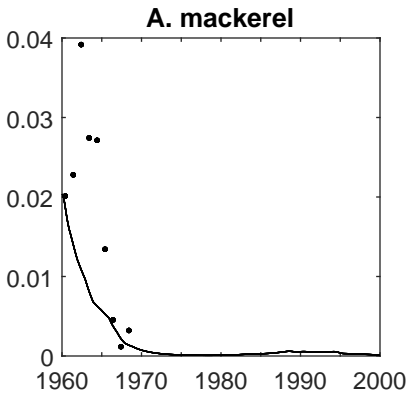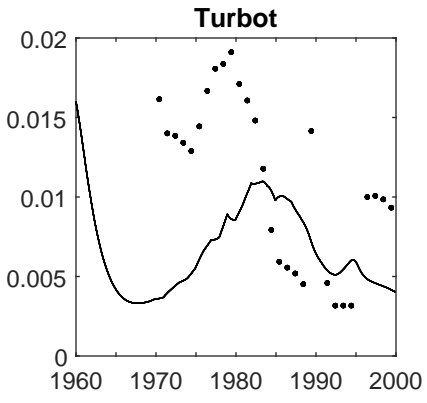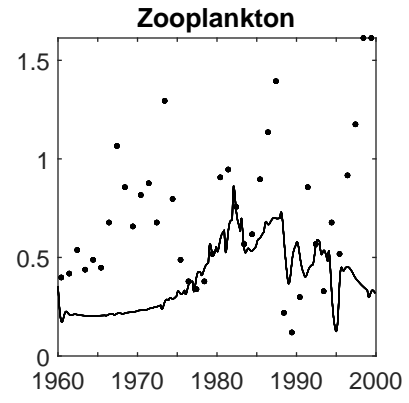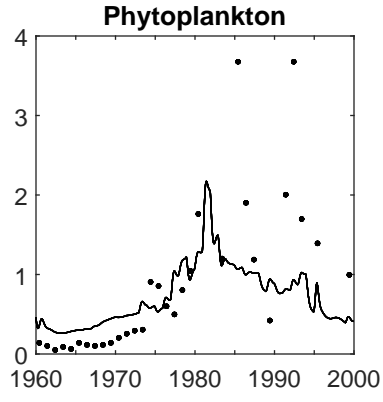

Supplement: Supplemental Information 6 [file peerj-11-15649-s006.pdf]

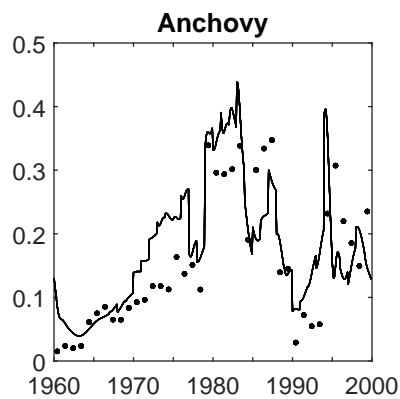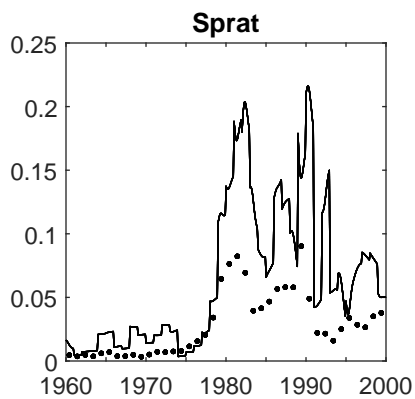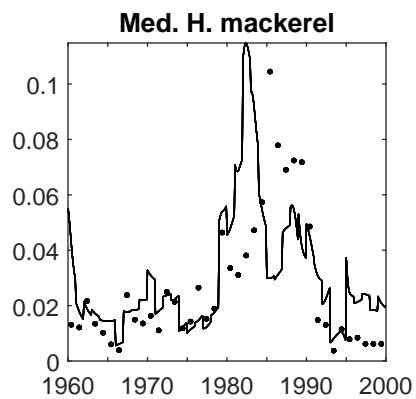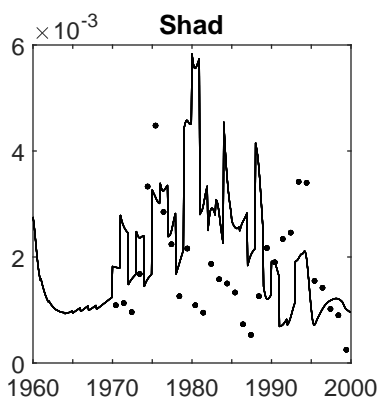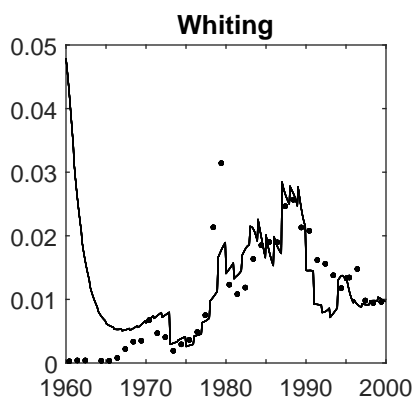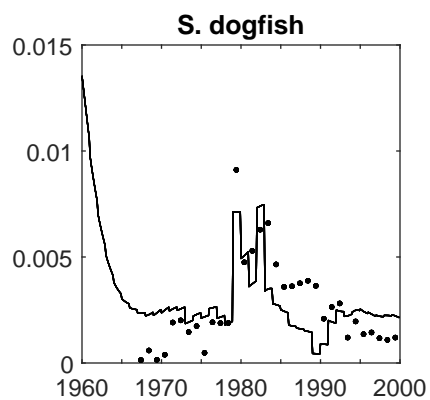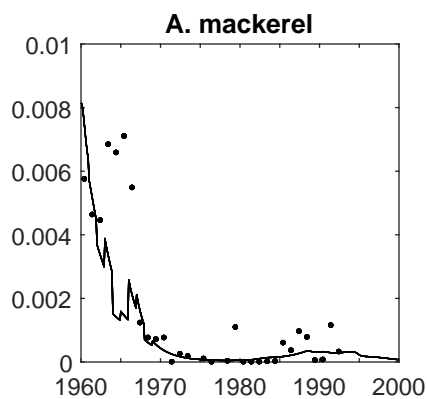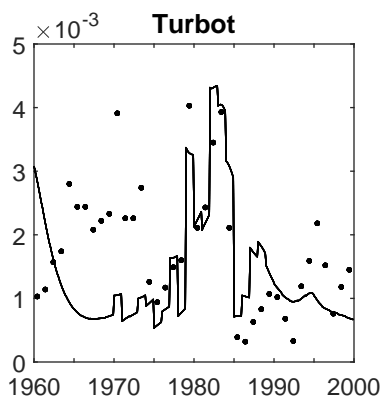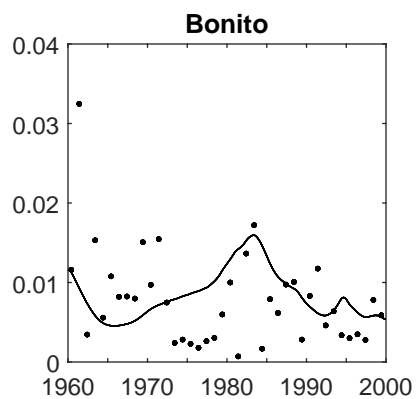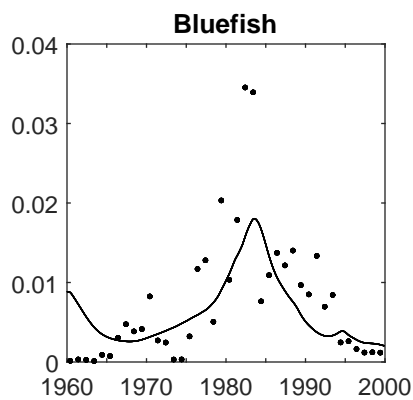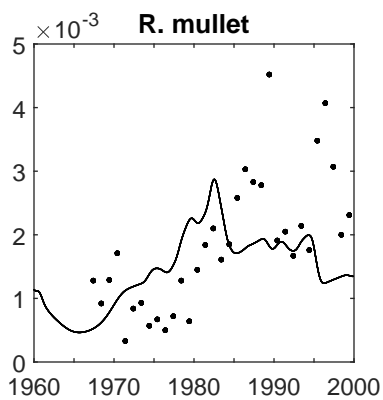

Supplement: Supplemental Information 7 [file peerj-11-15649-s007.pdf]

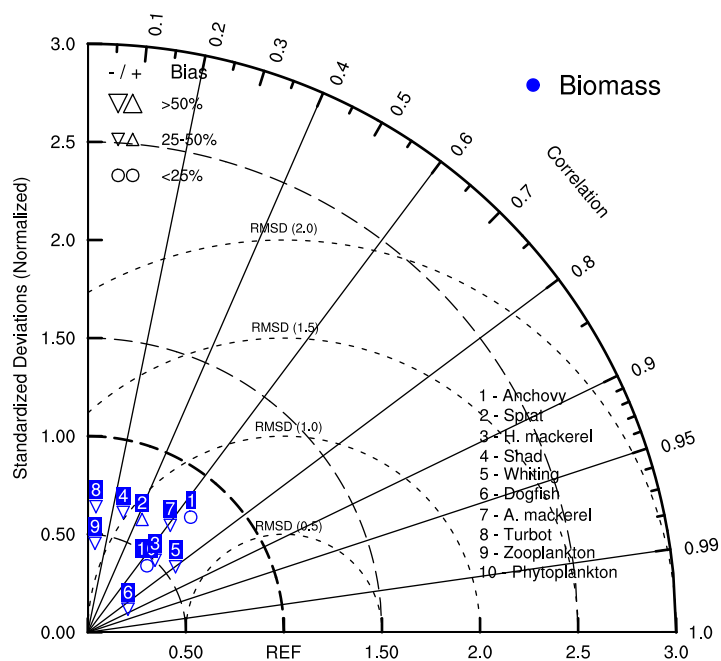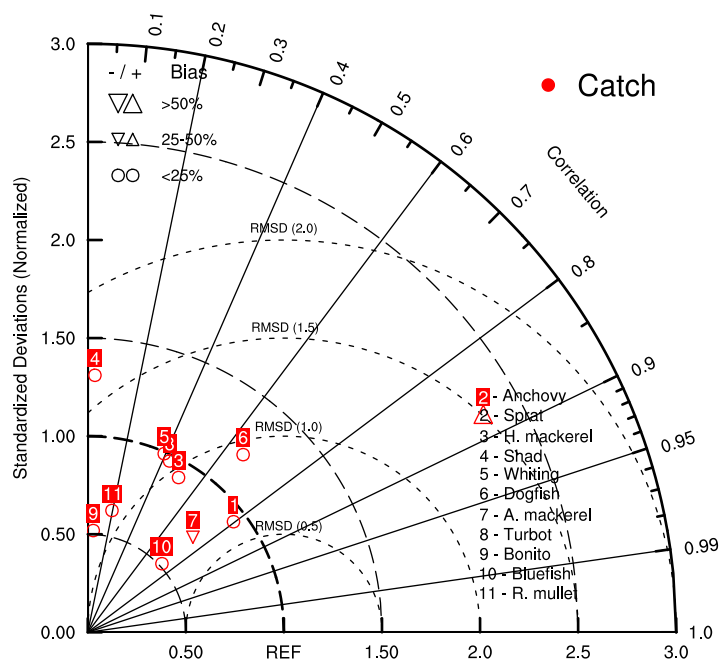

Supplement: Supplemental Information 8 — X and Y axes show standard deviations normalized in reference to the observations (REF on the X axis), the radial axis shows correlation coefficients of the model-simulated biomasses and catches to the XSA stock assessment and in-situ biomass and statistical catch values, and the dashed semi-circles show root mean square distance (RMSD) of the model-simulated values to the observations (REF). The skill of the model in reproducing the reference data increases towards the REF point on the X axis. Relative biases are shown with circles and triangles and the magnitude of the biases are proportional to the size of the shapes. [file peerj-11-15649-s008.pdf]

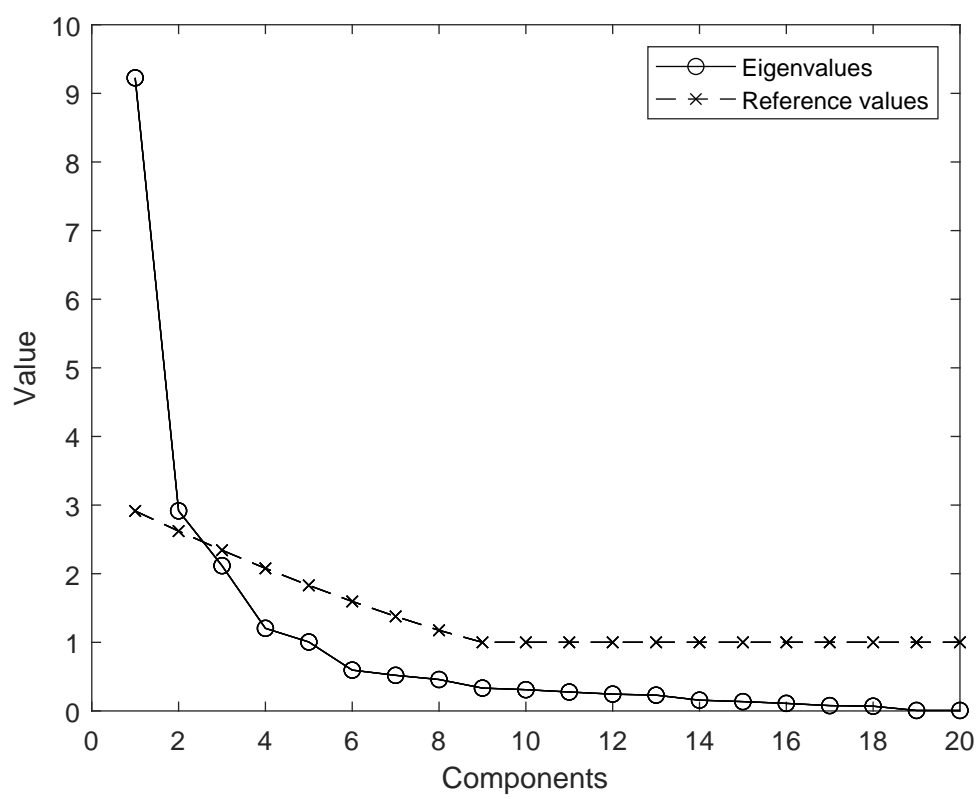

Supplement: Supplemental Information 9 — Principal components with eigenvalues that are greater than their corresponding reference values are retained considering the empirical Kaiser criterion. [file peerj-11-15649-s009.pdf]
